# Prevalence and duration of common symptoms in people with long COVID: a systematic review and meta-analysis

**DOI:** 10.7189/jogh.15.04282

**Published:** 2025-10-17

**Authors:** Siyi Luo, Lana Yh Lai, Rui Zhu, Yudong Gao, Zhenxi Zhao

**Affiliations:** 1 Department of Acute Infectious Disease Prevention and Control, Kunming Center for Disease Control and Prevention, Kunming, Yunnan, China; 2 School of Health Sciences, University of Manchester, Manchester, UK; 3 Shaanxi Nuclear Industry 215 Hospital, Clinical Laboratory, Xianyang, Shaanxi, China

## Abstract

**Background:**

During the COVID-19 pandemic, an increasing number of patients have reported persistent symptoms after recovery, a phenomenon known as long COVID. These symptoms may persist for weeks or months, affecting the patient's daily life and health. To systematically understand the long-term impact of long COVID, this study conducted a systematic review and meta-analysis. This study aims to determine the long-term effects of long COVID by identifying, evaluating and summarising the incidence and duration of persistent symptoms after the acute phase of COVID-19.

**Method:**

We searched PubMed, Embase, and medRxiv up to August 2021 for articles and preprints presenting original research on the symptoms of long COVID. Following title/abstract and full-text screening, based on the PICOS framework, we excluded articles that did not clearly report on diagnoses, reported on symptoms lasting less than four weeks, lacked epidemiological data, or did not provide complete data. We assessed the bias of included studies using the Newcastle-Ottawa Scale. For effects reported in more than two studies, we performed meta-analysis of prevalence, and also counted the duration of each symptom.

**Results:**

We included 19 observational studies in the meta-analysis, through which we determined the incidence and duration of five common long COVID symptoms, including cognitive/memory/attention disorders (36%, unreported duration), fatigue (34%, 5.5 months), mental health problems (including anxiety and depression, 31%, 3.5–3.8 months), and dyspnoea (24%, 6.52 months) and chest pain (23%, 2 months).

**Conclusions:**

The symptoms of long COVID usually persist after the acute phase of COVID-19. The clustering of long COVID symptoms provides a direction for studying the aetiology, diagnosis, and management of post-COVID conditions. Our findings provide important baseline data for the prevention and treatment of long COVID.

The outbreak of the COVID-19 pandemic led to approximately two million deaths in 2020 [1]. By January 2024, there were 774 million diagnoses and 6.96 million deaths reported worldwide [2]. COVID-19 is a multi-systemic, multi-organ disease, with multiple clinical manifestations [3]. While it mainly impacts the respiratory system in the acute phase, the disease also affects other organs such as the heart, kidneys, liver, muscles, and skin [4]. Testing and diagnostic criteria are divided into clinical and laboratory diagnoses, with the former being made by clinicians based on computerised tomography (CT) imaging findings or clinical symptoms, and the latter including nucleic acid testing (NAT) such as reverse transcription polymerase chain reaction (RT-PCR) and serum antigen-antibody testing [5]. Diagnostic modalities are increasingly evolving as the current state of infection of the disease is studied and regulated. Based on clinical diagnosis and follow-up, the incubation period of the COVID-19 virus in humans is typically 1–14 days, although this has been known to extend up to 24 days [6].

At that period, it is generally accepted that the recovery period for mild symptoms is 7–10 days in the acute phase of infection and 3–6 weeks in severe cases [7]. However, a follow-up of some patients showed that symptoms persist and recur for weeks or months after the acute phase [8]. Researchers have used the term ‘long COVID’ to refer to the condition where individuals who recovered from acute COVID-19 still report lasting effects of the infection, have a longer-than-expected clinical presentation, or even present new symptoms [9]. Due to a lack of understanding and constantly emerging evidence, early definitions of long COVID were sensitive rather than specific. Following a Delphi consensus exercise performed in October 2021, the World Health Organization (WHO) defined this condition as one affecting individuals with a history of probable or confirmed SARS-CoV-2 infection. It typically begins around three months after the onset of COVID-19, with symptoms lasting for at least two months that cannot be explained by any other diagnosis. These symptoms may emerge after initial recovery or persist from the initial illness, and they can fluctuate or recur over time [10]. Crook and colleagues [11] highlighted that long COVID could involve multiple organs and affect many systems, while Sudre and colleagues [12] found it was characterised by symptoms of fatigue, headache, shortness of breath, and anosmia, and reported a positive correlation between the likelihood of developing the condition and increasing age or body mass index.

Until mid-2021, significant progress has been made in the prevention, diagnosis, treatment, and management of COVID-19, owing to extensive and collaborative research efforts across the global scientific community. Sisó-Almirall and colleagues [13] observed that patients with long COVID-19 should be managed using structured primary care visits based on the time from diagnosis of SARS-CoV-2 infection. However, our lack of understanding of the long-term effects of this condition means that the duration and ideal frequency of follow-up have not yet been clearly defined.

The lack of a uniform classification and range of post-acute symptoms is another issue that needs to be tackled. Based on a descriptive systematic assessment, the symptoms of long COVID range from single manifestations such as fatigue, shortness of breath, and cough, to long-term systemic disorders such as pulmonary, cardiac, and neurological sequelae [14,15]. While some studies have reported on the symptoms of long COVID, we still lack a standardised methodology for defining and measuring these symptoms, as well as robust, comparable prevalence estimates for common symptoms. Michelen and colleagues [16] called for standardised and controlled studies into the aetiology, risk factors, and biomarkers to characterise long COVID in different at-risk populations and settings. Due to the lack of clear statistics on the incidence of symptoms, there is also a lack of guidelines for the treatment and evaluation of patients with long COVID. Therefore, further research and a comprehensive understanding of the recovery time, signs, or clinical disease of the long-term effects of COVID-19 is needed.

An initial scoping literature review found that studies of long COVID were mainly descriptive in nature, highlighting a lack of higher-level evidence such as meta-analyses [17]. To bridge this gap, we performed a systematic review and meta-analysis to determine and synthesise data on the common symptoms of long COVID and their duration.

## METHODS

We followed the PRISMA guidelines in reporting the findings of this systematic review [18].

### Inclusion criteria

Our inclusion criteria were based a modified PICOS framework, without specifying a comparator. Specifically, the study population were patients with confirmed COVID-19, defined by laboratory testing (*e.g.* PCR or serology) or clinical diagnosis. Exposures/interventions included all documented symptoms, whether self-reported or clinically identified. The primary outcome was the prevalence of specific long COVID symptoms, with a particular focus on the most frequently reported symptoms across studies. We included journal articles and preprints published between January 2020 and August 2021 presenting original data from epidemiological observational studies, or more specifically, prospective cohort and retrospective cohort studies and cross-sectional studies.

### Exclusion criteria

We excluded patients without any laboratory confirmation or clinical diagnosis who self-identified as having a COVID-19 infection or suspected infection, those not formally identified as having long COVID, and those with a symptom duration shorter than four weeks. The last exclusion criterion was based on several considerations. First, several authoritative organisations definition (such as the US Centers for Disease Control and Prevention, the WHO, *etc.*) usually define long COVID as a condition whose symptoms persist for weeks to months after COVID-19 infection [19,20]. By excluding research that describes short-term symptoms, we maintain consistency between the review goals and these definitions. Second, given that the acute symptoms of COVID-19 infection usually appear in the first few weeks after infection and may gradually diminish, excluding studies with symptoms lasting less than four weeks can provide a clearer distinction between those occurring in the acute phase and long-term COVID sequelae. Third, research on long COVID aims to explore long-term health problems after a COVID-19 infection and their mechanisms, which makes excluding studies focussed on short-term symptoms a sensible choice. Besides this, we excluded studies with symptom classification, but no data on prevalence or duration; studies that reported overall disease prevalence data, but not data on specific individual symptoms; and studies without full texts available, conference proceedings, and protocols.

### Search strategy and screening process

We developed a search strategy for PubMed and Embase using keywords and MeSH terms according to our PICOS framework (Table S1 in the **Online Supplementary Document**), which we subsequently adapted for MedRxiv to specifically identify studies that referenced ‘long COVID’ in their titles or abstracts. We exported all records into Endnote, version X9 (Clarivate, London, UK) for deduplication, after which two researchers (SL and YG) screened their titles and abstracts in duplicate, followed by the full-texts of any records remaining after this stage. Discrepancies were resolved in discussion with a third reviewer (ZZ). We also retrieved existing systematic reviews and cross-checked their reference lists with the studies identified through our primary search. Any articles that met the inclusion criteria through this reference search, but were missed during the initial retrieval, were manually added to the final list of included studies.

### Data collection and extraction

The two reviewers (SL and YG) independently extracted the following into a pre-designed sheet: study information (author, year, region and title), methodology, population characteristics, time frame, COVID-19 assay, primary symptoms. Regarding the outcome of interest, we extracted data on sample size (denominator), number of symptoms (numerator), prevalence (percentage, frequency, prevalence), follow-up time (mean (standard deviation)/median (interquartile range)), and duration of symptoms (mean/median/interquartile range). Symptoms were categorised according to a predefined systemic classification adapted from Cabrera Martimbianco and colleagues [21], which includes respiratory, digestive, mental health, neurological, and other symptoms, while vaguely defined or unclassifiable symptoms were grouped under ‘other symptoms’ (**Table 1****)**. Discrepancies at this stage were resolved in discussion with a third researcher (ZZ).

**Table 1:** Classification of symptoms

System	Symptoms
Neurological	Fatigue, cognitive/memory/attention disorders, sleep disorder, taste disorders, olfactory disorders
Respiratory	Shortness of breath, chest distress, cough, influenza symptoms (fever)
Digestive	Diarrhoea
Psychological/mental health	Anxiety, depression, somnipathy
Other symptoms	Loss of appetite, weight loss, pain (muscle/joint), activity barriers, post-activity polypnoea, sweating, problems finding words

### Assessment for risk of bias

Two reviewers (SL and GY) independently assessed all included studies for risk of bias using the Newcastle-Ottawa Scale (NOS) for nonrandomised studies [22] (Table S2 in the **Online Supplementary Document**), resolving discrepancies through discussion. The NOS focusses on three main areas: selection of participants, determination of exposure/outcome, and comparability of the groups involved, where a study can be given a maximum of one star for each numbered item within the ‘Selection’ and ‘Outcome’ categories, and two stars for ‘Comparability’. Cohort studies can be given a maximum of nine stars, with those receiving scores of five and above being considered high quality. The assessment of comparability and outcomes included the assignment of stars if the study adjusted for important confounding factors, such as age. A follow-up period exceeding one month was considered sufficient for outcome assessment and was awarded one star. An additional star was awarded if the follow-up rate exceeded 80% and reasons for loss to follow-up were appropriately described, indicating adequate cohort retention.

The adapted quality assessment scale for cross-sectional studies permitted a maximum of eight stars. Studies attaining a score of five or more stars were deemed high quality, whereas those with four or fewer were categorised as low quality

### Data analysis

We summarised the data using descriptive statistics, presenting them as frequencies and percentages. We performed the meta-analysis for the main and generated forest plots for the prevalence and duration of symptoms of long COVID using *R*, version 4.1.0 (R Foundation for Statistical Computing, Vienna, Austria) and the ‘metaprop’ [23] package. We handled missing data by calculating the number of prevalent cases using available percentages and sample sizes. Based on the assumptions that COVID-19 has long-term effects that vary across studies and that the study populations were heterogeneous, we used a random effect meta-analysis, analysing proportions on the logit scale and presenting estimates as 95% confidence intervals (CIs), with significance set at *P* < 0.05 and heterogeneity assessed using *I*^2^ statistics.

Meanwhile, the durations were summarised by Microsoft Excel (Microsoft Corporation, Redmond, WA, USA) and show the average duration of symptoms by system. We estimated the standard deviation of the time using descriptive statistics to ensure the completeness of the central tendency estimation.

### Assessment of heterogeneity

We assessed the studies for heterogeneity using the *I*^2^ statistics by Higgins and Thompson [24] and by inspecting forest plots. The interpretation of heterogeneity was based on the *I*^2^ statistic, using the following conventional thresholds: 0–40% represents low heterogeneity, where the study is homogeneous; 30–60% represents moderate heterogeneity; 50–90% represents substantial heterogeneity; 75–100% represents high heterogeneity, where studies are not homogeneous. The *I*^2^ statistic was interpreted using conventional thresholds where overlapping ranges (*e.g.* 30–60% for moderate) reflect gradual transitions rather than distinct categories. 

## RESULTS

### Study selection process

**Figure 1:**
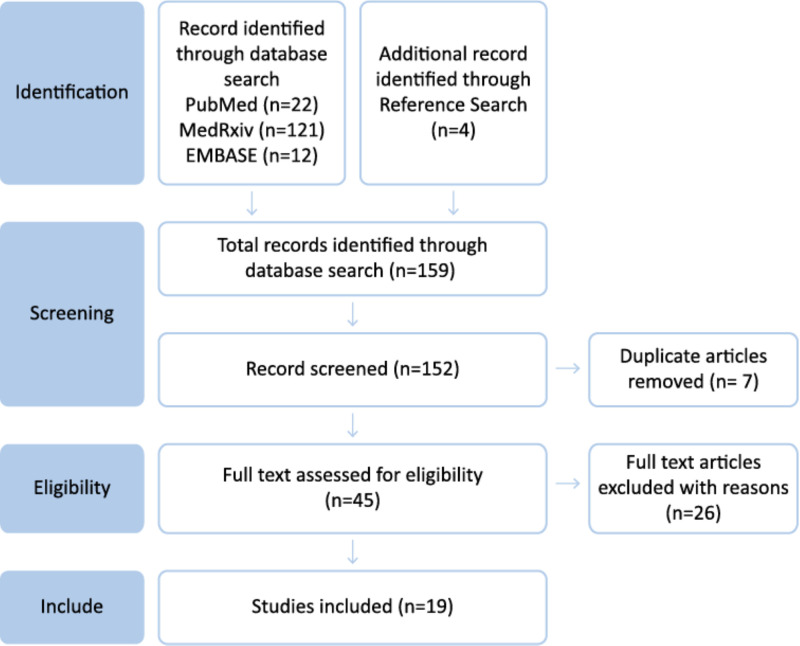
PRISMA flow diagram

Our search retrieved 159 publications, with 152 remaining after deduplication; 107 were excluded during title/abstract screening and a further 26 during full-text evaluation (**Figure 1**). A total of 34 articles from the references were rigorously screened for full text, of which four studies met the criteria (all from long COVID descriptive reviews) and were therefore included. We identified 19 articles as meeting the inclusion criteria in the final review, with 15 originating from database searches and 4 manually added from reference searches (Table S3 in the **Online Supplementary Document**).

### Characteristics of the included studies

Eleven studies (58%) were performed in Europe (three from Italy, two from France and Germany, six from the UK, Denmark (including the Faroe Islands), and Ireland), seven in Asian countries (two studies in China, one each in Iran, Russia, Pakistan, Turkey, and India), and only one in the USA. Regarding their participants, two studies recruited children, while all others recruited adults over 18 years of age or people of all ages. In terms of gender, the study populations were predominantly female, accounting for 41–72.2% of their samples. Five studies used both a positive laboratory test and a clinical diagnosis to confirm COVID-19. Fifteen articles used laboratory PCR testing to confirm COVID-19 infection, of which five reported confirmation through both a positive laboratory test and a clinically confirmed diagnosis.

### Quality assessment

All articles included in the review were critically assessed through the NOS scale (**Table 2**). The cohort studies received NOS scores ranging from 4–7 stars, with a mean of 4.8. Three studies scored four stars and were therefore judged to be of low quality [25,28,35]. Most of the follow-ups in the outcome evaluation were longer than one month or had more than one month between follow-ups, with only one study not meeting this condition [37] and therefore did not receive a score in the ‘results’ section. All articles were identified as confirming COVID-19 exposure, meaning they had no non-exposed group. The NOS scores for the cross-sectional studies ranged from four to six stars, with a mean of 5.3. Only one study was given a low methodological quality rating [43].

**Table 2:** Quality assessment of included studies

	Selection				Comparability		Outcome			Total *	Risk of bias level
	**NOS1**	**NOS2**	**NOS3**	**NOS4**	**NOS5**	**NOS6**	**NOS7**	**NOS8**	**NOS9**		
**Cohort studies**											
Augustin, 2021 [25]	*				*		*	*		4/9	Low
Boscolo-Rizzo, 2021 [26]	*		*	*	*		*	*	*	7/9	High
Huang, 2021 [27]	*		*	*	*		*	*		6/9	High
Mirfazeli, 2021 [28]	*		*				*	*		4/9	Low
Osmanov, 2021 [29]	*		*			*	*	*	*	6/9	High
Scherlinger, 2021 [30]	*		*			*	*	*	*	6/9	High
Boscolo-Rizzo, 2021 [31]	*		*	*	*		*	*	*	7/9	High
Miskowaik, 2021 [32]	*		*		*	*	*	*	*	7/9	High
Petersen, 2020 [33]	*		*		*			*	*	5/9	High
Seeßle, 2021 [34]	*		*			*		*	*	5/9	High
Kayaaslan, 2021 [35]	*		*			*			*	4/9	Low
Budhiraja, 2021 [36]	*		*			*		*	*	5/9	High
Huang, 2021 [37]	*		*		*	*		*	*	6/9	High
Carvalho-Schneider, 2020 [38]	*		*		*		*	*	*	6/9	High
Xiong, 2021 [39]	*		*		*			*	*	5/9	High
**Cross-sectional studies**											
Buonsenso, 2021 [40]	*	*		*			*	*		5/8	High
Ziauddeen, 2021 [41]	*	*		*	*	*		*		6/8	High
Townsend, 2020 [42]	*			*	*	*	*	*		6/8	High
Iqbal, 2021 [43]	*	*		*					*	4/8	Low

NOS – Newcastle-Ottawa scale

Eleven of the 19 studies adjusted for their significant confounders [25,26,31–33,37–39,40–42], nine adjusted for any potential confounders, including length of follow-up, confirmation of diagnosis and mode of testing [29,30,32,34–37,41,42], while three did not adjust for confounding factors and therefore had no score in terms of ‘Comparability’ [29,28,40].

### Classification and prevalence of common symptoms

**Figure 2:**
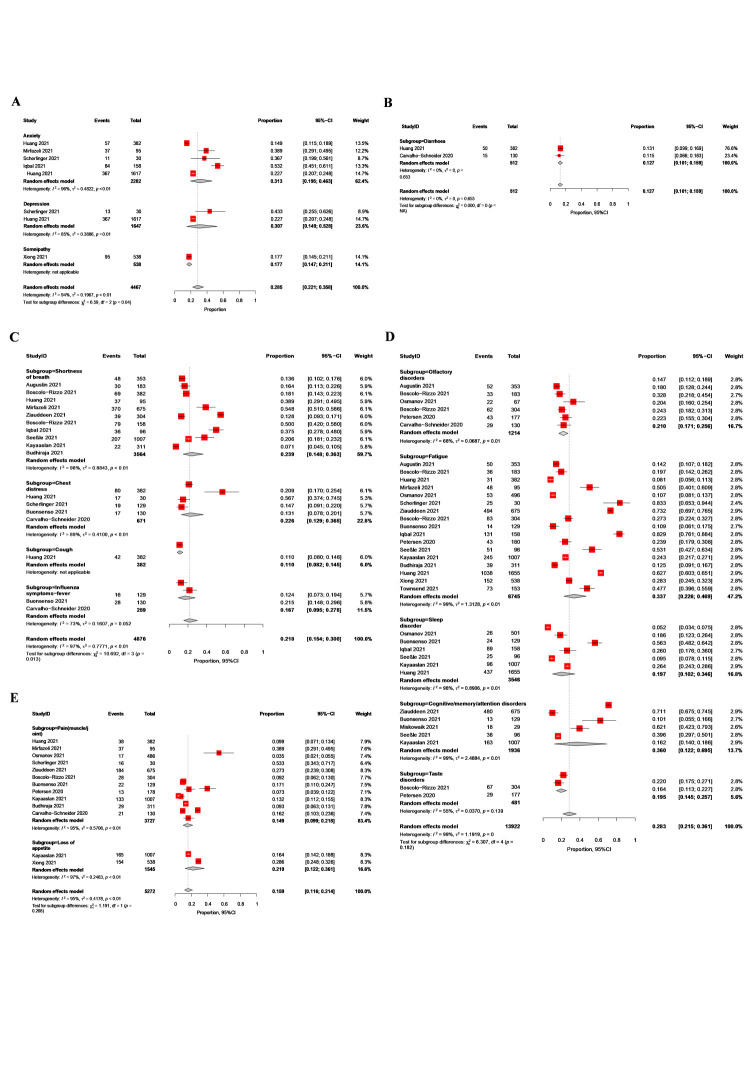
Forest plot of the prevalence (proportion) of common symptoms in the system. **Panel A.** Mental health. **Panel B.** Digestive system. **Panel C.** Respiratory system. **Panel D.** Nervous system. **Panel E.** Pain and loss of appetite.

Eight disorders from six studies were selected for inclusion in this system for mental health [27–29,37,39,43], including anxiety, depression, and somnipathy. There was no difference in the prevalence of depression and anxiety (*P* = 0.950) (**Figure 2**, Panel A). The prevalence in the two studies on the digestive system with 512 study participants [27,38] did not differ significantly (*P* = 0.653) (**Figure 2**, Panel B), and it had 12.7% of diarrhoeal symptoms (*I*^2^ = 0%).

Thirteen studies reported on respiratory-related symptoms [25–28,30,31,34–36,40,41,43] (**Figure 2**, Panel C). Shortness of breath carried the highest weight in the pooled estimate, indicating it had the greatest influence on the overall result (weighting of 59.7%). Chest distress and shortness of breath had a similar likelihood of prevalence, but studies reporting on the former had slightly less heterogeneity than those reporting on the latter (*I*^2^ = 98%), suggesting they were more consistent in their findings. Only one study reported an 11% probability of persistent cough. Two studies reported persistent symptoms of influenza [26,38], with 16–17% individuals suffering from fever-like symptoms.

Nineteen articles reported on neurological symptoms (**Figure 2**, Panel D), with fatigue being most commonly reported and accounting for a high weight of effects (47.2%). The most common neurological symptoms of long COVID were fatigue and cognitive/memory/attention impairment, followed by olfactory dysfunction. Moreover, sleep disorders and taste disorders had a similar prevalence. There was no significant difference in prevalence among the neurological subgroups (*P* = 0.182).

Heterogeneity was lower in the subgroup of studies reporting taste disorders (*I*^2^ = 55%), followed by those reporting olfactory disorders (*I*^2^ = 68%). The highest heterogeneity was observed among studies reporting fatigue and cognitive/memory/attention disorders (*I*^2^ = 99%). Heterogeneity may be attributed to the small sample size in one study on fatigue [37] and the large prevalence ratio reported in another study within the same subgroup by Iqbal and colleagues [26]. Similarly, in the cognitive/memory/attention disorders subgroup, two studies presented significantly divergent prevalence estimates compared to the rest of the included studies [30,34].

Twelve studies reported on other symptoms [27–30,33–36,38,39], with pain (defined as muscle pain or joint pain) being the most frequently reported one (**Figure 2**, Panel E). However, the prevalence of loss of appetite was higher than that of pain, although this difference was not significant (*P* = 0.268). Two of the studies included in the meta-analysis showed similar prevalence in terms of pain (proportions ranging from 0.092 to 0.093) [26,36]. The remaining symptoms in this category, such as activity barriers, problems finding words, weight loss, sweating, and post-activity polypnoea, were not meta-analysed, as they were reported in single studies only [34,38,39].

### Exploring heterogeneity

Most studies included in the subgroup analysis had high heterogeneity. Only the study on digestion was more consistent (*I*^2^ = 0%). According to the effect size distribution, weights were more balanced under the random effects model, with studies with small sample sizes being weighted more heavily and studies with large sample sizes being weighted relatively less heavily [30,43]. As we included studies from different countries and populations, the random effects model allowed some of this dispersion to reflect actual differences in effect sizes across regions.

### Duration of symptoms

**Figure 3:**
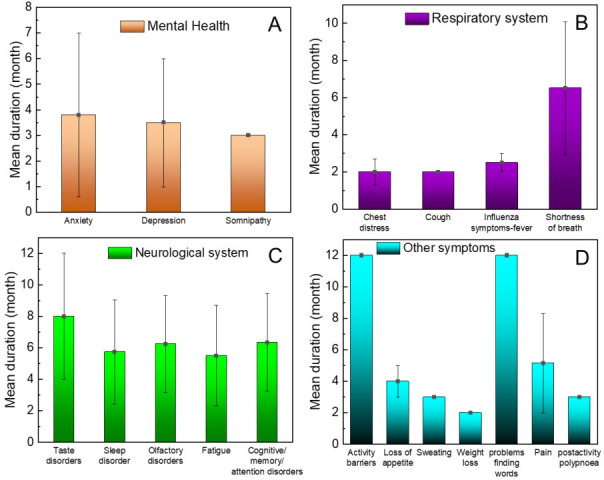
Duration of symptoms. **Panel A.** Mental health. **Panel B.** Respiratory symptoms. **Panel C.** Neurological symptoms. **Panel D.** Other symptoms.

In terms of mental health impact, anxiety symptoms lasted the longest in individuals with long COVID, with a mean duration of 3.8 months. Depression and somnipathy had durations of 3.5 and 3 months, respectively (**Figure 3**, Panel A). Both studies that included the digestive system showed the same duration of diarrhoea, at 2 months. The duration of symptoms in COVID-19 post-acute sequalae ranged from 2 months to a mean of 6.52 months (**Figure 3**, Panel B). Symptoms of cough and chest distress persisted until 2 months after diagnosis. Shortness of breath emerged as the symptom with the most long-term effects in terms of the respiratory system, with a mean duration of 6.52 months across 10 studies (**Figure 3**, Panel C**)**. Long-term manifestations of sleep disturbance (5.57 months), olfactory disorders (6.25 months), fatigue (5.5 months) and cognitive impairment (6.34 months) (including memory and attention impairment) were present for approximately 5–6 months after the COVID-19 infection. Taste disorders were present for an average of 8 months after infection. The duration of other symptoms ranged from 2 months to 1 year (**Figure 3**, Panel D**)**.

## DISCUSSION

### Summary of main findings

Here we identified 15 cohort studies and 4 cross-sectional studies that explored the common symptoms of long COVID and their duration. Most of the studies confirmed COVID-18 through laboratory tests (PCR) and were conducted in non-hospitalised patients. We identified 20 long-term symptoms grouped under five major systems. Neurological symptoms were the most frequent, being reported in 19 studies, while digestive symptoms were found least frequently, in two studies only. Approximately 13–28% of patients experienced long-term persistent symptoms 2–12 months after diagnosis, including fever, sore throat, cough, muscle or body pain, loss of taste or smell, and diarrhoea, similar to those seen in the acute phase of COVID-19 [44].

In terms of the duration, activity disorders and word finding difficulties were the longest-lasting symptoms (12 months). Chest distress, cough, and weight loss had a shorter duration of impact at 2 months. The average duration of the remaining five most common disorders ranged from 3.5 months to 6.52 months.

### Interpretation of findings and relation to existing work

Several descriptive systematic evaluations [21,45] and rapid evaluations [46] assessing the long-term effects of COVID-19 have similarly identified fatigue, chest distress, shortness of breath, and reduced mental and cognitive status as the most commonly reported symptoms. However, prevalence estimates, symptoms and duration definitions differed slightly from those we observed here.

Anxiety (31%, 3.8 months) and depression (31%, 3.5 months) were found to be the most common disorders in the mental health system, with similar prevalence of both symptoms. A four-month qualitative study of 114 patients with long COVID showed that mental health was a persistent and recurrent symptom after acute COVID-19 [47]. A three-month study by van den Borst and colleagues found that 31% of participants (24 of 76 assessed patients) had abnormal psychological questionnaire scores, based on the Hospital Anxiety and Depression Scale measure [48]. The abnormalities in mental health seem likely to be explained by patients' doubts about their ability to regain their health and concerns about stigma. Respondents in the first of the above-mentioned studies doubt about recovery from long COVID due to the presence of numerous and frequently recurring symptoms and an uncertain prognosis [47]. Many of the study narratives conveyed feelings of blame and shame for having COVID-19 [49], and participants described feelings of disconnection and isolation experienced in trying to access service support [47].

Diarrhoea was the only symptom reported for the digestive system (13%, 2 months). Sudre and colleagues [50] identified gastrointestinal symptoms and persistent fever as a multisystem disorder lasting up to 58 days. However, few studies reported this symptom to be common. A detailed overview of long COVID concluded that the gastrointestinal syndrome after COVID-19 consisted mainly of abdominal discomfort, diarrhoea, constipation, and vomiting [51]. This may be related to the effects of the medication taken during acute COVID-19, with individuals taking lopinavir/ritonavir, which produces gastrointestinal symptoms [52]. They are therefore thought to be a possible residual symptom after acute COVID-19 [51]. However, more research is needed on gastrointestinal symptoms in this context due to a paucity of studies.

Regarding the respiratory system, the main common symptoms identified here were chest distress (23%) and shortness of breath (24%), with an average duration of 2–6.5 months. A follow-up study in China found that 66% of patients with mild to moderate symptoms still had abnormal CT images at three months post-discharge [53]. A comprehensive health assessment and CT imaging of patients within three months of COVID-19 found that over 90% of patients discharged had abnormal lung parenchyma, which was associated with reduced lung diffusion capacity [48]. The reduced diffusing capacity of the lungs is reflected in a reduced diffusing capacity of the lungs for carbon monoxide, leading to hypoxic conditions with compromised oxygen saturation and consequent shortness of breath, cyanosis of the lips, and even panic and chest distress [54,55]. Lopez-Leon and colleagues reported the prevalence of shortness of breath to be 24% [55], which aligns with our study. Notably, outcome assessment in the included studies rarely included imaging, with most outcomes being self-reported, meaning there were no CT findings or standard assessment of lung function recovery. Therefore, symptoms associated with pulmonary dysfunction may not be comprehensive in terms of structural and functional abnormalities, requiring additional investigation of long-term respiratory sequelae.

For symptoms related to the nervous system, a study focusing on fatigue reported a high prevalence of 52.3% in the post-acute phase, demonstrating that it was a common sequelae regardless of the initial disease severity; at a median follow-up of 72 days post-symptom onset, over half of the participants (52.3%) still experienced persistent fatigue [56]. In this review, the duration of fatigue (34%) averaged 5.5 months. In comparison, another study focussing on the symptoms of fatigue after COVID-19 showed that one third of patients were accompanied by clinical manifestations of fatigue for up to one year [57]. This could be explained by the fact that only one study included in this review had a follow-up of up to one year [27]. Others have described severe incapacitating fatigue, pain, neurocognitive impairment, sleep deprivation and impairment of cognitive activity as chronic fatigue syndrome lasting longer than six months [58]. However, the joint psychiatric and neurological definition of chronic fatigue syndrome has been contested, as the causative factors of CFS are not clearly defined and may be related to viral infections, immune dysfunction, endocrine metabolic dysfunction, or neuropsychiatric factors [59]. Here we categorised these symptoms separately to facilitate a clearer comparison of the prevalence of symptoms belonging to the neurological and psychiatric systems. The neurological symptoms reported here also included cognitive/memory/attention impairment (36%), loss of smell (21%), and loss of taste (20%), while studies by other researchers rarely describe loss of taste and/or smell [60]. Although symptoms of fatigue, pain, and activity impairment overlap with the long-term symptoms of severe acute respiratory syndrome [61], loss of taste and/or smell has not been described in studies of post-infection syndromes of diseases other than COVID-19. Lu and colleagues found that patients with COVID-19 were more likely to have abnormalities in brain regions, including those associated with loss of smell and memory, compared to non-infected individuals [62]. Another study concluded that this is likely to be a symptom that is specific to long COVID [60].

Here, we reported loss of appetite (22%, 4 months) was the most common symptom in the ‘other’ category, followed by pain (including headache and chest pain, 15%, 5 months). The remaining symptoms in other systems (difficulties finding words, sweating, post-activity polyuria, *etc.*) were reported only once and were likely to be incidental. Headache was also considered as a persistent symptom in some studies, with 10% of outpatients reporting headaches lasting 13 weeks [63]. Few studies considered loss of appetite to be a common symptom. This may be due to the different classifications of the definition of the system here compared to other studies.

### Interpretation of heterogeneity

The reasons for the high level of heterogeneity could not be fully explored, as we did not conduct further subgroup analyses for age, gender, or hospitalisation/discharge. However, we hypothesise that the observed heterogeneity may be related to differences in measurement methods, duration of follow-up, and the inclusion of different populations with different characteristics. For example, some of the included studies were based on the patients’ self-reporting, while others used formal assessment tests. Self-reporting by patients may exaggerate reality due to their greater concern about the disease. Similarly, the different thresholds (*e.g.* binary or 0–10 scale) used to determine adverse outcomes in each study may have led to differences in prevalence. Furthermore, the different follow-up time periods for short-term measures (1 month) and long-term (12 months) may affect prevalence estimates across studies. The third source of heterogeneity could be related to the characteristics of the included populations, namely inpatients, discharged patients and outpatients. The severity of disease for long COVID varied between time periods. In the digestive system, we observed a high level of concordance between the two included studies [27,38], which may be related to the fact that they only included inpatients. The higher reliability of hospital-based diagnoses and laboratory confirmation in these inpatient cohorts further strengthens the consistency of the findings. However, these findings are derived from a limited number of studies (n = 2) conducted exclusively among inpatient populations and may lack generalisability to the broader long COVID cohort documented in other studies (incorporate non-hospitalised and community-based cases). This requires further separate subgroup analysis for each stage of the disease and the classification of inpatients in the acute phase as ICU/non-ICU with reference to the inclusion criteria.

### Implications for public health practice and future research

This study supports the development of clinical practice guidelines and informs innovative policy by providing an updated classification of long COVID symptoms into five major systems encompassing 19 manifestations, including mental health, respiratory, neurological, and other symptoms, along with their prevalence. This enables general practitioners to identify and manage these conditions more efficiently. It also offers guidance on optimal follow-up duration and frequency for outpatients and recovering patients, supplies up-to-date educational resources for healthcare professionals and students, and highlights the need to focus on ambulatory patients with mild to moderate infection who require ongoing medical and social support. For public health practice, the findings underscore the importance of clinicians monitoring mental health, neurological, and respiratory symptoms [64], managing common long-term issues such as anxiety, depression, fatigue, and breathlessness [65], and providing psychological interventions during hospitalisation [66]. Furthermore, healthcare institutions and departments should raise awareness of long COVID among both professionals and the public, emphasising its long-term impacts beyond the acute phase of illness.

Our findings highlight that further clarity is needed to establish baseline characteristics of long COVID, including age, past history of symptoms and severity of COVID-19. This is also true for the changes of long COVID in different groups of people over time. Further research is needed to identify a set of potentially relevant risk factors for the disease. Due to the continued prevalence of COVID-19, and as new research emerges, systematic review should continue to be periodically updated to fully define a standard set of symptoms, duration, and temporal criteria for the acute and post-acute phases of belonging to long COVID.

### Limitations

This study does have some limitations. The first is related to the different naming and definition of symptoms in the post-acute phase of COVID-19 in the included studies and their diagnosis according to different criteria. Furthermore, much of the heterogeneity observed here resulted from differences in baseline characteristics across studies, such as differences in age groups, prior history of COVID-19, and differences in the severity of long COVID. The included cohort studies lacked controls, and we could not determine whether the onset of symptoms was due to COVID-19. In particular, symptoms related to mental health may emerge due to a variety of factors, such as hospitalisation, socioenvironmental factors during the pandemic or the effects of long-term treatment. The studies included in our review enrolled participants encompassing mild, moderate, and severe cases without stratifying them by disease severity or follow-up time, thereby precluding detailed stage-specific analysis. We did not consider long-term symptom trajectories beyond the acute phase, limiting our understanding of the ongoing effects of COVID-19, particularly in distinguishing whether symptoms represent persistent sequelae or newly emerging conditions. Moreover, not all studies pre-defined long-term symptoms and lacked systematic reporting of symptoms that resolved during the follow-up period.

Additional limitations are the inadequate sample sizes in the included studies and the limited generalisability of our results. Specifically, we included studies with small sample sizes (<100 participants) that were predominantly conducted in Europe and Asia, with only one study conducted in the USA. There were also some variations across studies in the selection of subjects and how their outcomes were measured, including the use of unvalidated measures and instruments to assess outcomes (questionnaires). Self-reporting – a method used in several of the studies – is also subject to recall and response bias. Lastly, although we considered preprints in our meta-analysis, we note that they are not peer-reviewed and are therefore subject to unpredictable errors. Such risk could be mitigated by searching institutional or other databases that have quality filters not present in preprint servers.

## CONCLUSIONS

In this review, we identified and synthesised 19 observational studies published from January 2020 to August 2021, providing evidence on persistent symptoms that occur after COVID-19 infection, *i.e.* in relation to the condition known as long COVID. Our findings show that these symptoms mainly include prolonged fatigue, cognitive/memory/concentration impairment, chest distress, and breathlessness. Our findings could inform the development of clinical guidelines, such as those for symptom monitoring and follow-up protocols, and support policy efforts that see to allocate resources toward high-burden sequelae like mental health, respiratory, and neurological conditions. The widespread and sustained impact of long COVID underscores the need for evidence-based responses. 

## Additional material


Online Supplementary Document
