# Supplementary material for: Prevalence and duration of common symptoms in people with long COVID: a systematic review and meta-analysis
Source: J Glob Health. 2025 Oct 17;15:04282. doi: 10.7189/jogh.15.04282 (PMC12532441; doi:10.7189/jogh.15.04282)
Supplement: Online Supplementary Document [file jogh-15-04282-s001.zip › jogh-15-04282-s001.pdf]

Online Supplementary

Table S1. Search terms for PubMed&EMBASE (Ovid)

Database	Search Terms	Query	Records retrieved
PubMed	Population:<br>#1patient	patient* OR participant*	8,560,913 results
	Exposure:<br>#2long COVID	(long covid) AND ((long term) OR (long-haulers) OR complication* OR post OR chronic)	321 results
	Outcome:<br>#3symptom	symptom* OR syndrom* OR diagnos*	6,978,430 results
	#4Prevanlence	preval* OR inciden* OR occur* OR survey*	4,813,747 results
	#5duration	duration* OR *period* OR frequenc*	1,508,191 results
	Combine	#1，#2，#3，#4 AND #5	22 results
Embase(Ovid)	Population:<br>#1patient	patient* OR participant*	8128279 results
	Exposure:<br>#2long COVID	(long covid) AND ((long term) OR (long-haulers) OR complication*)	231 results
	Outcome:<br>#3symptom	symptom* OR syndrom* OR diagnos*	6789741 results
	#4Prevanlence	preval* OR inciden* OR occur* OR survey*	4649362 results
	#5duration	duration* OR *period* OR frequenc*	16141851 results
	Combine	#1，#2，#3，#4 AND #5	12 results

Table S2: Assessment of risk and bias

New-Castle Ottawa Quality Assessment Scale For Cohort studies

(A study can be awarded a maximum of one star for each numbered item within the Selection and Outcome categories. A maximum of two stars can be given for Comparability)

**Content of the assessment**	Questions	**records ✱**
**Selection(Maximum of 1 star for each numbered item /Maximum of 4 stars in total)**	1) <u>Representativeness of the exposed cohort</u><br>a)Truly representative of the population infected with Covid19 (described) in the community ❋<br>b) somewhat representative of the average in the community ❋<br>c) selected group of users eg nurses, volunteers<br>d) no description of the derivation of the cohort<br>2) <u>Selection of the non exposed cohort</u><br>a) drawn from the same community as the exposed cohort ❋<br>b) drawn from a different source<br>c) no description of the derivation of the non exposed cohort<br>3) <u>Ascertainment of exposure</u><br>a) secure record (eg surgical records) ❋<br>b) structured interview ❋<br>c) written self report<br>d) no description<br>4) <u>Demonstration that outcome of interest was not present at start of study</u><br>a) yes ❋<br>b) no	

**Comparability (Maximum of 2 stars in total)**	1) <u>Comparability of cohorts on the basis of the design or analysis</u><br>a)study controls for (ages/sex/Follow-up date/confirmation of diagnosis/testing method) (select the most important factor) ❋<br>b) study controls for any additional factor ❋	
**Outcome(Maximum of 1 star for each numbered item/Maximum of 3 stars)**	1) <u>Assessment of outcome</u><br>a) independent blind assessment ❋<br>b) record linkage ❋<br>c) self report<br>d) no description<br>2) <u>Was follow-up long enough for outcomes to occur</u><br>a) yes(>1months) (select an adequate follow up period for outcome of interest) ❋<br>b) no<br>3) <u>Adequacy of follow up of cohorts</u><br>a) complete follow up - all subjects accounted for ❋<br>b) subjects lost to follow up unlikely to introduce bias - small number lost - > __20__ % (select an adequate %) follow up, or description provided of those lost) ❋<br>c) follow up rate < __80__% (select an adequate %) and no description of those lost<br>d) no statement	
	Total： /9	

New-Castle Ottawa Quality Assessment Scale For Cross-sectional studies

	Questions	✱

**Selection: (Maximum 4 stars)**	1) <u>Representativeness of the sample:</u><br>a) Truly representative of the average in the target population. ∗ (all subjects or random sampling)<br>b) Somewhat representative of the average in the target population. ∗ (non-random sampling)<br>c) Selected group of users.<br>d) No description of the sampling strategy.<br><br>2) <u>Sample size:</u><br>a) Justified and satisfactory. ∗<br>b) Not justified.<br><br>3) <u>Non-respondents:</u><br>a) Comparability between respondents and non-respondents characteristics is established, and the response rate is satisfactory. ∗<br>b) The response rate is unsatisfactory, or the comparability between respondents and non-respondents is unsatisfactory.<br>c) No description of the response rate or the characteristics of the responders and the non-responders.<br><br>4) <u>Ascertainment of the exposure (risk factor):</u><br>a) Validated measurement tool. ∗<br>b) Non-validated measurement tool, but the tool is available or described.∗<br>c) No description of the measurement tool.	
**Comparability: (Maximum 2 stars)**	1) <u>The subjects in different outcome groups are comparable, based on the study design or analysis. Confounding factors are controlled.</u><br>a) The study controls for the most important factor (age). ∗<br>b) The study control for any additional factor. ∗	
	1) <u>Assessment of the outcome:</u><br>a) Independent blind assessment. ∗	

**Outcome:**<br>**(Maximum 2 stars)**	b) Record linkage. ✱<br>c) Self report.<br>d) No description<br><br>2) <u>Statistical test:</u><br>a) The statistical test used to analyze the data is clearly described and appropriate, and the measurement of the association is presented, including confidence intervals and the probability level (p value). ✱<br>b) The statistical test is not appropriate, not described or incomplete.	
	Total： /8	

Supplementary Table S3: Characteristics of included studies

Study ID	Publication year & title	Country	Study design	Study population					
				No. participants	Age (IQR/median/range/mean)	Sex	Participant characteristics	Time horizon/data recruitment dates	Criteria for diagnosis
Augustin 2021 [1]	Long-term health consequences after mild COVID in non-hospitalized patients	Germany	Prospective cohort study	985	43 (IQR31-54)	513 female	non-hospitalized	6th April to 2nd December 2020	laboratory diagnosis
Boscolo-Rizzo 2021[2]	High prevalence of long-term psychophysical olfactory dysfunction in patients with COVID-19	Italy	Prospective cohort study	202	median age 55 (IQR21-84)	80 female	non-hospitalized	NA	laboratory diagnosis

Buonsenso 2021[3]	Preliminary Evidence on Long COVID in children	Italy	Cross-secti onal studies	129 children	mean age11 ± 4.4 years	62 (48.1%) female	hospitalized & non-hospitalized	March to November, 2020	clinical diagnosis
Huang 2021[4]	COVID Symptoms, Symptom Clusters, and Predictors for Becoming a Long-Haule r: Looking for Clarity in the Haze of the Pandemic	United States	Prospective cohort study	1407	NA	653 male (46%) 754 female (54%)	non-hospitalized	2nd April 2021	laboratory diagnosis

Mirfazeli 2021[5]	Acute phase clinical manifestation of COVID-19 linking to long-COVID symptoms	Iran	Prospective cohort study	201	median age 50 (range 28-86)	58% male (n = 55)	52 non-hospitalized /149 hospitalized	March 2019 to April 2020	laboratory diagnosis & clinical diagnosis
Osmanov 2021[6]	Risk factors for long covid in previously hospitalised children using the 1 ISARIC Global follow-up protocol: A prospective cohort study	Russia	Prospective cohort study	518 children	median age 10.4 years (IQR, 3-15.2; range, 2 days-18 years)	272 (52.2%) female	rehabilitation discharged/Recovered	2nd April 2020 to 26th August 2020	Laboratory diagnosis

Scherlinger 2021[7]	Refining “long-COVID” by a prospective multimodal evaluation of patients with long-term symptoms related to SARS-CoV-2 infection	France	Prospective cohort study	30	median age 40 (IQR 35-54)	18 (60%) were female	non-hospitalized	February to April 2020	clinical diagnosis
Ziauddeen 2021[8]	Characteristics of Long COVID: findings from a social media survey	United Kingdom	Cross-sectional studies	2550	median age 46.5 (SD 11years)	82.8% females	non-hospitalized	7th – 14th November 2020	clinical diagnosis
Boscolo-Rizzo 2021[9]	Long COVID In Adults at 12 Months After Mild-to-Moderate	Italy	Prospective cohort study	354	median age 47 (18-76) years	185 (60.9%) females	non-hospitalized	1st – 31st March 2020	laboratory diagnosis & clinical diagnosis

	SARS-CoV-2 Infection								
Miskowaik 2021[10]	Cognitive impairments four months after COVID-19 hospital discharge: Pattern, severity and association with illness variables	Denmark	Prospective cohort study	29	mean (SD) 56.2 (10.6)	females (%) 12 (41)	rehabilitation discharged/ recovered	June to November 2020	laboratory diagnosis & clinical diagnosis
Iqbal 2021[11]	The COVID-19 Sequelae: A Cross-Sectional Evaluation of Post-recovery Symptoms	Pakistan	Cross-sectional studies	158	mean age 32.10 ± 12.42 years (age range: 19-80 years)	55.1% females	rehabilitation discharged/ recovered	September 2020 to December 2020	laboratory diagnosis

	and the Need for Rehabilitation of COVID-19 Survivors								
Petersen 2020[12]	Long COVID in the Faroe Islands: A Longitudinal Study Among Non-hospitalized Patients	Faroe Islands	Prospective cohort study	180	mean (SD, range) age 39.9 years (19.4, 0-93)	54.4% female	hospitalized & non-hospitalized	22 April to 16 August 2020	laboratory diagnosis
Seeßle 2021[13]	Persistent symptoms in adult patients one year after COVID-19: a prospective cohort study	Germany	Prospective cohort study	146	Age median (IQR) 57 (50-63)	43 (44.8%) male	hospitalized & non-hospitalized	NA	laboratory diagnosis & clinical diagnosis

Kayaaslan 2021[14]	Post-COVID syndrome: A single-center questionnaire study on 1007 participants recovered from COVID-19	Turkey	Prospective cohort study	1007	mean age 45.0 ± 16.4 (18–88) and 39.9% were 50 years and older	54.4% male	hospitalized & non-hospitalized	December 2020 and February 2021	laboratory diagnosis & clinical diagnosis
Budhiraja 2021[15]	Long Term Health Consequences of COVID-19 in Hospitalized Patients from North India: A follow up study of up to 12 months	India	Prospective cohort study	990	range from 1-91 years	320 (32.3%) female	hospitalized	September 2020 to March 2021	laboratory diagnosis

Huang 2021[16]	6-month consequences of COVID-19 in patients discharged from hospital: a cohort study	China	ambidirectional cohort study	1733	median age of 57.0 (IQR 47.0–65.0) years	48% female	hospitalized	January to May 2020	laboratory diagnosis
Carvalho-Schneider 2020[17]	Follow-up of adults with noncritical COVID-19 two months after symptom onset	France	Prospective cohort study	150 adults	(≥18 years) Mean age (SD): 49 ±15 years	56% female	hospitalized & non-hospitalized	March to June 2020	laboratory diagnosis
Xiong 2021[18]	Clinical sequelae of COVID-19 survivors in Wuhan, China: a single-centre longitudinal study	China	Retrospective cohort study	538 survivors	the median (IQR) age was 52.0 (41.0–62.0) years	293, 54.5% female	rehabilitation discharged/ recovered	NA	clinical diagnosis

Townsend 2020[19]	Persistent Poor Health PostCOVID-19 Is Not Associated with Respiratory Complications or Initial Disease Severity	Ireland	Cross-sectional studies	153 (Admitted non-ICU N = 55, ICU N = 19, Non-admitted, N = 79)	non-ICU Age - 56.4 years, ICU Age - 54.5 years, Non-admitted Age - 40.2 years	Admitted non-ICU (15.5) 47.3% female, ICU (11.6) 26.3% female, Non-admitted (11.4) 72.2% female	hospitalized & non-hospitalized	March to May 2020	laboratory diagnosis

Supplement Reference:

1. Augustin M, Schommers P, Stecher M, Dewald F, Gieselmann L, Gruell H, et al. Recovered not restored: long-term health consequences after mild COVID-19 in non-hospitalized patients. medRxiv. 2021 Mar 15:[preprint]. doi:10.1101/2021.03.11.21253207

2. Boscolo-Rizzo P, Menegaldo A, Fabbris C, Spinato G, Borsetto D, Vaira LA, et al. High prevalence of long-term psychophysical olfactory dysfunction in patients with COVID-19. medRxiv. 2021 Jan 8:[preprint]. doi:10.1101/2021.01.07.21249406

3. Huang Y, Pinto MD, Borelli JL, Mehrabadi MA, Abrihim H, Dutt A, et al. COVID symptoms, symptom clusters, and predictors for becoming a long-hauler: looking for clarity in the haze of the pandemic. Clin Nurs Res. 2022;31(8):1390-8. doi:10.1177/10547738221095251

4. Mirfazeli FS, Sarabi-Jamab A, Kordi A, Abolhasani Foroughi A, Riahi A, Dahi R, et al. Acute phase clinical manifestation of COVID-19 is linked to long-COVID symptoms: a 9-month follow-up study. medRxiv. 2021 Jul 16:[preprint]. doi:10.1101/2021.07.13.21260482

5. Osmanov IM, Spiridonova E, Bobkova P, Gamirova A, Shikhaleva A, Andreeva M, et al. Risk factors for post-COVID-19 condition in previously hospitalised children using the ISARIC global follow-up protocol: a prospective cohort study. Eur Respir J. 2022;59(2):2101341. doi:10.1183/13993003.01341-2021

6. Scherlinger M, Felten R, Gallais F, Szabados C, Salmon JH, Meyer A, et al. Refining “long-COVID” by a prospective multimodal evaluation of patients with long-term symptoms related to SARS-CoV-2 infection. Infect Dis Ther. 2021;10(3):1747-63. doi:10.1007/s40121-021-00491-x

7. Boscolo-Rizzo P, Guida F, Polesel J, Marcuzzo AV, Capriotti V, D’ Alessandro D, et al. Long COVID in adults at 12 months after mild-to-moderate SARS-CoV-2 infection. medRxiv. 2021 Apr 15:[preprint]. doi:10.1101/2021.04.12.21255343

8. Miskowiak KW, Johnsen S, Sattler SM, Nielsen S, Kunalan K, Rungby J, et al. Cognitive impairments four months after COVID-19 hospital discharge: pattern, severity and association with illness variables. Eur Neuropsychopharmacol. 2021;46:39-48. doi:10.1016/j.euroneuro.2021.03.019

9. Petersen MS, Kristiansen MF, Hanusson KD, Danielsen ME, á Steig B, Gaini S, et al. Long COVID in the Faroe Islands: a longitudinal study among non-hospitalized patients. Clin Infect Dis. 2021;73(11):e4058-63. doi:10.1093/cid/ciaa179

10. Seeßle J, Waterboer T, Hippchen T, Spix A, Müller B, Merle U. Persistent symptoms in adult patients 1 year after coronavirus disease 2019 (COVID-19): a prospective cohort study. Clin Infect Dis. 2022;74(7):1191-8. doi:10.1093/cid/ciab611

11. Kayaaslan B, Eser F, Kalem AK, Kaya G, Kaplan B, Kaya G, et al. Post-COVID syndrome: a single-center questionnaire study on 1007 participants recovered from COVID-19. J Med Virol. 2021;93(12):6566-74. doi:10.1002/jmv.27156

12. Budhiraja S, Aggarwal M, Wig R, Ramakrishnan N, Kabi A, Agarwal M, et al. Long term health consequences of COVID-19 in hospitalized patients from north India: a follow up study of up to 12 months. medRxiv. 2021 Jun 23:[preprint]. doi:10.1101/2021.06.21.21258543

13. Huang L, Li X, Gu X, Zhang H, Ren L, Guo L, et al. 6-month

consequences of COVID-19 in patients discharged from hospital: a cohort study. Lancet. 2021;398(10298):220-32. doi:10.1016/S0140-6736(21)00368-9

14. Carvalho-Schneider C, Laurent E, Lemaignen A, Beaufils E, Bourbao-Tournois C, Laribi S, et al. Follow-up of adults with noncritical COVID-19 two months after symptom onset. Clin Microbiol Infect. 2021;27(2):258-63. doi:10.1016/j.cmi.2020.09.041

15. Xiong Q, Xu M, Li J, Liu Y, Zhang J, Xu Y, et al. Clinical sequelae of COVID-19 survivors in Wuhan, China: a single-centre longitudinal study. Clin Microbiol Infect. 2021;27(1):89-95. doi:10.1016/j.cmi.2020.09.023

16. Buonsenso D, Munblit D, De Rose C, Sinatti D, Ricchiuto A, Carfi A, et al. Preliminary evidence on long COVID in children. Acta Paediatr. 2021;110(7):2208-11. doi:10.1111/apa.15870

17. Ziauddeen N, Gurdasani D, O' Hara ME, Hastie C, Roderick P, Yates T, et al. Characteristics of long COVID: findings from a social media survey. medRxiv. 2021 Mar 24:[preprint]. doi:10.1101/2021.03.21.21253968

18. Townsend L, Dowds J, O’ Brien K, Sheill G, Dyer AH, O’ Kelly B, et al. Persistent poor health after COVID-19 is not associated with respiratory complications or initial disease severity. Ann Am Thorac Soc. 2021;18(6):997-1003. doi:10.1513/AnnalsATS.202009-1175OC

19. Iqbal A, Iqbal K, Ali SA, Iqbal F, Iqbal Z. The COVID-19 sequelae: a cross-sectional evaluation of post-recovery symptoms and the need for rehabilitation of COVID-19 survivors. Cureus. 2021;13(2):e13080. doi:10.7759/cureus.13080
